# Comparison of transcriptomes of an orthotospovirus vector and non-vector thrips species

**DOI:** 10.1371/journal.pone.0223438

**Published:** 2019-10-10

**Authors:** Anita Shrestha, Donald E. Champagne, Albert K. Culbreath, Mark R. Abney, Rajagopalbabu Srinivasan

**Affiliations:** 1 Department of Entomology, University of Georgia, Griffin, GA, United States of America; 2 Department of Entomology, University of Georgia, Athens, GA, United States of America; 3 Department of Plant Pathology, University of Georgia, Tifton, GA, United States of America; 4 Department of Entomology, University of Georgia, Tifton, GA, United States of America; University of Idaho, UNITED STATES

## Abstract

Thrips transmit one of the most devastating plant viruses worldwide–tomato spotted wilt tospoviru*s* (TSWV). *Tomato spotted wilt tospovirus* is a type species in the genus *Orthotospovirus* and family *Tospoviridae*. Although there are more than 7,000 thrips species, only nine thrips species are known to transmit TSWV. In this study, we investigated the molecular factors that could affect thrips ability to transmit TSWV. We assembled transcriptomes of a vector, *Frankliniella fusca* [Hinds], and a non-vector, *Frankliniella tritici* [Fitch], and performed qualitative comparisons of contigs associated with virus reception, virus infection, and innate immunity. Annotations of *F*. *fusca* and *F*. *tritici* contigs revealed slight differences across biological process and molecular functional groups. Comparison of virus cell surface receptors revealed that homologs of integrin were present in both species. However, homologs of another receptor, heperan sulfate, were present in *F*. *fusca* alone. Contigs associated with virus replication were identified in both species, but a contig involved in inhibition of virus replication (radical s-adenosylmethionine) was only present in the non-vector, *F*. *tritici*. Additionally, some differences in immune signaling pathways were identified between vector and non-vector thrips. Detailed investigations are necessary to functionally characterize these differences between vector and non-vector thrips and assess their relevance in orthotospovirus transmission.

## Introduction

Thrips-transmitted tomato spotted wilt tospovirus (TSWV) ranks among the ten most detrimental plant viruses worldwide [1]. *Tomato spotted wilt tospovirus* is the type species of the genus *Orthotospovirus* in the family *Tospoviridae* and order *Bunyavirales*. TSWV is an enveloped single-stranded ambisense RNA virus that is transmitted exclusively by thrips in a persistent and propagative manner [2–5]. Of the thousands of thrips species known worldwide, nine species alone are known to transmit TSWV, and only fourteen species in total are documented to transmit all known orthotospoviruses [6–8]. Also, all known vector thrips species are confined to the suborder Terebrantia and family Thripidae [9]. Several speculations have been made as to why some thrips species are vectors and others are not, but conclusive explanation or evidence is still lacking [10–11].

The interactions between thrips vectors and TSWV are complex. For instance, thrips exhibit stage-specific acquisition and inoculation of the virus. The virus must be acquired at the first or the second instar larval stage for successful inoculation at the adult stage. If thrips acquire the virus for the first time as adults, they will not be able to inoculate the virus. Once ingested, TSWV travels through the foregut to the midgut where it replicates and is subsequently translocated into salivary glands for further replication. During this process, TSWV crosses several membrane barriers [12, 13]. The complexity of TSWV-thrips interactions is further enhanced by the fact that the exact route of the virus from the midgut to salivary glands is unknown. Three hypotheses explaining the mechanism of TSWV translocation into the salivary glands have been proposed, including movement through a temporary ligament connecting the midgut and salivary glands formed during the early larval stages, through hemocoel, and through direct virus movement facilitated by proximity between the salivary glands and midgut tissues during the early larval stages [11–15]. Of the three hypotheses, the one suggesting virus movement through the ligament is supported, as studies have demonstrated salivary gland infection following TSWV infection in the ligament structure [11, 16]. The temporary ligament connecting the midgut to salivary glands is formed during the larval stages. However, as larvae develop, the connection is believed to be lost [11, 14]. Thus, only thrips that acquire TSWV during the early larval stages can serve as TSWV vectors. It is unknown whether the ligament connecting midgut tissues to salivary glands is present in all thrips species. Studies indicate that very closely related thrips species within the same genus, and presumably with a similar anatomy, function as vectors and non-vectors [10, 15,16]. Therefore, it would be reasonable to assume that ligament may not be the ultimate determinant of orthotospovirus transmission by thrips.

Few studies have investigated non-vector thrips species to elucidate factors determining thrips inability to serve as TSWV vectors [9, 10]. Assis Filho *et al*. (2005) demonstrated that in a non-vector thrips species, eastern flower thrips, *Frankliniella tritici* [Fitch], TSWV replicated successfully in the midgut epithelial cells. However, TSWV infection in the salivary glands was completely absent. Translocation of TSWV from the midgut to the salivary glands is a requisite for TSWV transmission. It is likely that the lack of TSWV in salivary glands despite successful accumulation in midgut cells is due to the midgut escape barriers, or suppression of TSWV replication caused by rapid degradation of virions in the midgut [17]. Insects possess highly efficient innate immunity that degrades and inhibits pathogen replication [18–21]. Insects’ innate immunity includes immune genes that recognize conserved motifs of invading pathogens termed pathogen associated molecular patterns (PAMP) [22, 23]. Once pathogens such as viruses are recognized, extracellular cascades are activated to amplify different signals, which lead to systemic production of pathogen suppressing molecules like antimicrobial peptides that ultimately degrade pathogens [24–26]. Until now, differences between vector and non-vector thrips species in terms of their innate immunity have not been explored. Comparative studies investigating the immune genes present in thrips species could help understand whether vector and non-vector thrips species vary in their innate immunity.

For successful virus-vector interactions, virus receptors must facilitate recognition and entry of viruses into host cells [27]. TSWV entry into thrips cells requires binding of TSWV glycoproteins to receptors located in the epithelial cells of thrips midgut. A 50-kDa protein has been identified as a putative receptor of TSWV in thrips [28]. However, that protein has not been characterized. TSWV is one of the few plant-infecting viruses in bunyavirales. The glycoprotein envelope of TSWV shares a similar structure and motif as that of other animal-infecting members of bunyavirales [29]. Several receptors that interact with the glycoproteins of animal-infecting members of bunyavirales have been identified. For instance, receptors including integrin, nucleolin, heparan sulfate, and dendritic cell-specific intercellular adhesion molecule-3-grabbing non-integrin (DC-SIGN) that facilitate entry of hantaviruses, nairoviruses, and phleboviruses have been identified [30–33]. Identifying similar receptors in thrips transcriptomes could provide insights into whether the presence of homologs of such receptors vary between vector and non-vector thrips species.

Comparative studies between vector and non-vector species at a molecular level could help identify species-specific constitutive factors that function as determinants of TSWV transmission. Recently, transcriptomes of different life stages of two main vector species: *Frankliniella fusca* (Hinds) and *Frankliniella occidentalis* (Pergande) were examined [34, 35]. However, transcriptomes of non-vector thrips species have neither been examined nor compared with the transcriptomes of vector thrips species. The main objective of this study was to compare transcriptomes of a vector (*F*. *fusca*) and a non-vector (*F*. *tritici*), and to investigate contigs (contiguous sequences) associated with virus-vector interactions including virus reception, virus infection, and innate immunity.

## Results

### Processing of the RNA-Seq reads and transcriptome assembly

Trimmomatic was used to process the sequencing reads. Trimming of the adapter sequences and filtering of low quality reads resulted in 36.6 million quality reads in *F*. *fusca* and 22.7 million high quality reads in *F*. *Tritici*. The clean reads were *de novo* assembled using Trinity into 27,025 and 23,605 contigs for *F*. *fusca* and *F*. *tritici*, respectively. N50 of the assembled contigs in *F*. *fusca* were 2,633 bases long, while the N50 of the assembled contigs in *F*. *tritici* was 2,975 bases “Table 1”. Further, evaluation of completeness of the *de novo* assemblies in *F*. *fusca* and *F*. *tritici* through the Core Eukaryotic Genes Mapping Approach (CEGMA) revealed that 99 and 100% of core proteins that are conserved within eukaryotes were present in *F*. *fusca* and *F*. *tritici*, respectively. BUSCO analysis revealed that 95.1 and 96.2% of 1658 single-copy gene orthologs from 42 insect species were present in *F*. *fusca* and *F*. *tritici* transcriptomes, respectively.

**Table 1:** Summary statistics of transcriptomes.

	*F*. *fusca*	*F*. *tritici*
**Total no. of clean reads**	36,554,797	22,788,837
**Total no. of assembled contigs**	27,025	23,605
**Mean contig length (bases)**	1,444	1,612
**Median contig length (bases)**	758	855
**N50 contig length (bases)**	2,633	2,975
**Total size of contigs**	39,024,499	38,040,196

Summary of *de novo* assembly of *Frankliniella fusca* and *Frankliniella tritici* transcriptomes using Trinity.

### Functional annotations of *F*. *fusca* and *F*. *tritici* contigs

**Fig 1:**
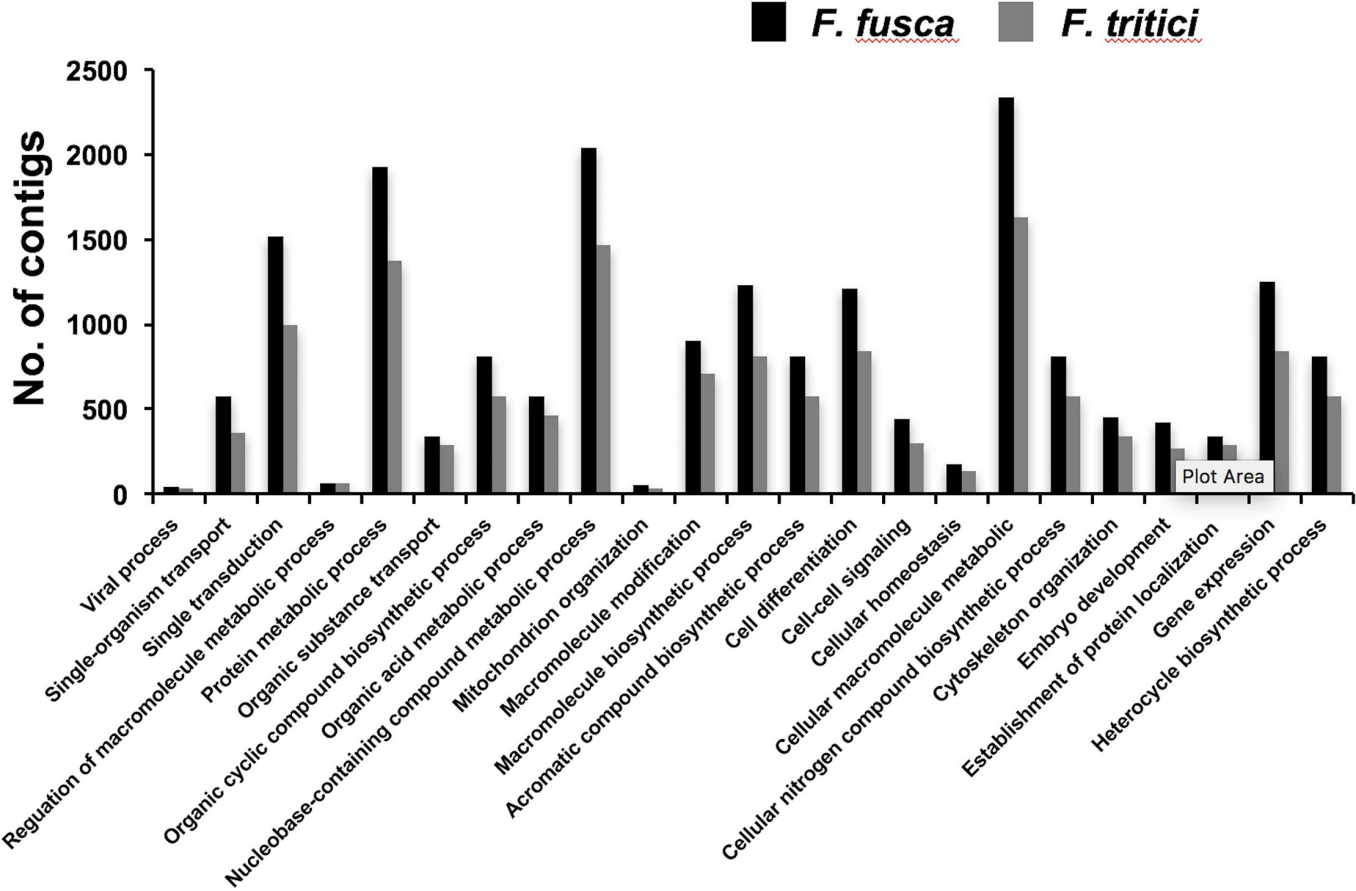
Gene ontology terms under the biological process category. Gene Ontology (GO) terms assigned to *Frankliniella fusca* and *Frankliniella tritici* under the biological process category using Blast2go analysis. GO terms were assigned to the contigs at level 5 with a node score of 5.

**Fig 2:**
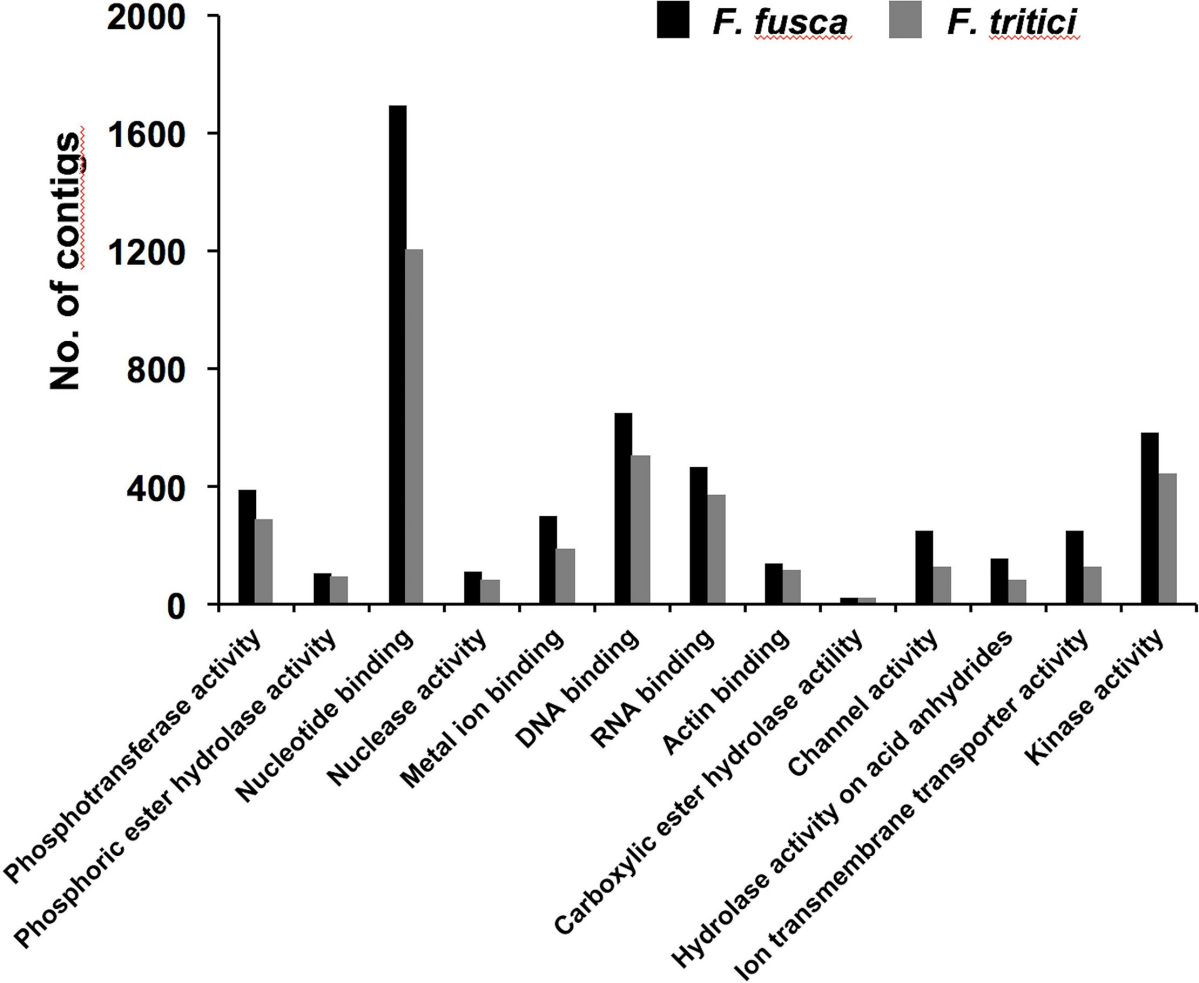
Gene ontology terms under the molecular function category. Gene Ontology (GO) terms associated with the molecular function category identified in *Frankliniella fusca* and *Frankliniella tritici* using Blast2go analysis. GO terms were assigned under the molecular function category at level 5 with a node score of 5.

**Fig 3:**
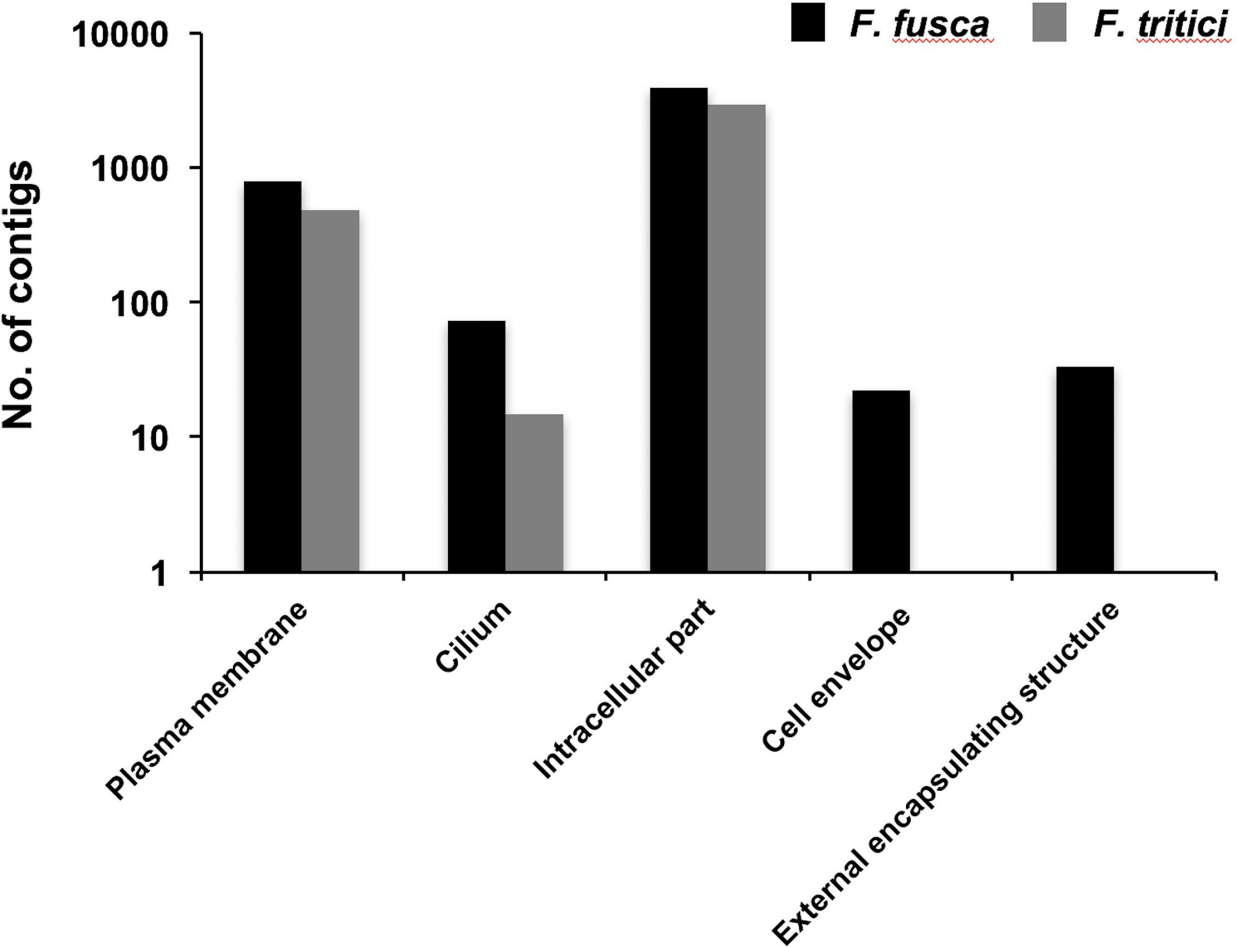
Gene ontology terms associated with the cellular component category. Gene Ontology (GO) terms associated with the cellular component category assigned to *Frankliniella fusca* and *Frankliniella tritici* by Blast2go analysis at level 5 with a node score of 5.

*Frankliniella fusca* and *F*. *tritici* contigs were annotated using Blastx search against NCBI non-redundant database. Blastx retrieved sequence match for 14,081 contigs in *F*. *fusca* and 12,984 contigs in *F*. *tritici*. Annotated contigs were further assigned functional groups under three classification systems using Blast2go analysis: biological process, molecular function, and cellular component. Blast2go assigned 23 Gene Ontology (GO) terms under the biological process category “Fig 1” and 13 GO terms under the molecular function category “Fig 2”. Most of the contigs under the biological process category consisted of functional annotations associated with cellular macromolecule metabolic process (12%), protein metabolic process (10%), nucleobase-containing compound metabolic process (10%), signal transduction (7%), and gene expression (6%). Under the molecular function category, nucleotide binding was the most dominant GO term (32%) followed by DNA binding (12%), kinase activity (12%), and phosphotransferase activity (7%). In both categories, all the GO terms that were assigned to *F*. *fusca* were also present in *F*. *tritici*. In the cellular component category, five and three GO terms were assigned to *F*. *fusca* and *F*. *tritici*, respectively “Fig 3”. Two GO terms specific to *F*. *fusca* included cell envelope and external encapsulating structure.

Pathway analysis mapped 127 and 125 biochemical pathways to *F*. *fusca* and *F*. *tritici*, respectively “S1 Table”. Most of the pathways were associated with metabolism including carbohydrate metabolism, biosynthesis of secondary metabolites, glycan biosynthesis and metabolism, lipid metabolism, and amino acid metabolism. Five pathways including toluene degradation, biotin metabolism, flavone and flavonol biosynthesis, PI3K-Akt signaling pathways, and nitrotoluene degradation were unique to *F*. *fusca*. However, three pathways associated with sesquiterpenoid/triterpenoid biosynthesis, vitamin B6 metabolism, and D-arginine and D-ornithine metabolism were unique to *F*. *tritici*.

Following the overview of functional annotation and pathway analyses of *F*. *fusca* and *F*. *tritici* contigs, contigs that could influence virus-vector interactions were examined.

### Virus receptors in *F*. *fusca* and *F*. *tritici*

**Fig 4:**
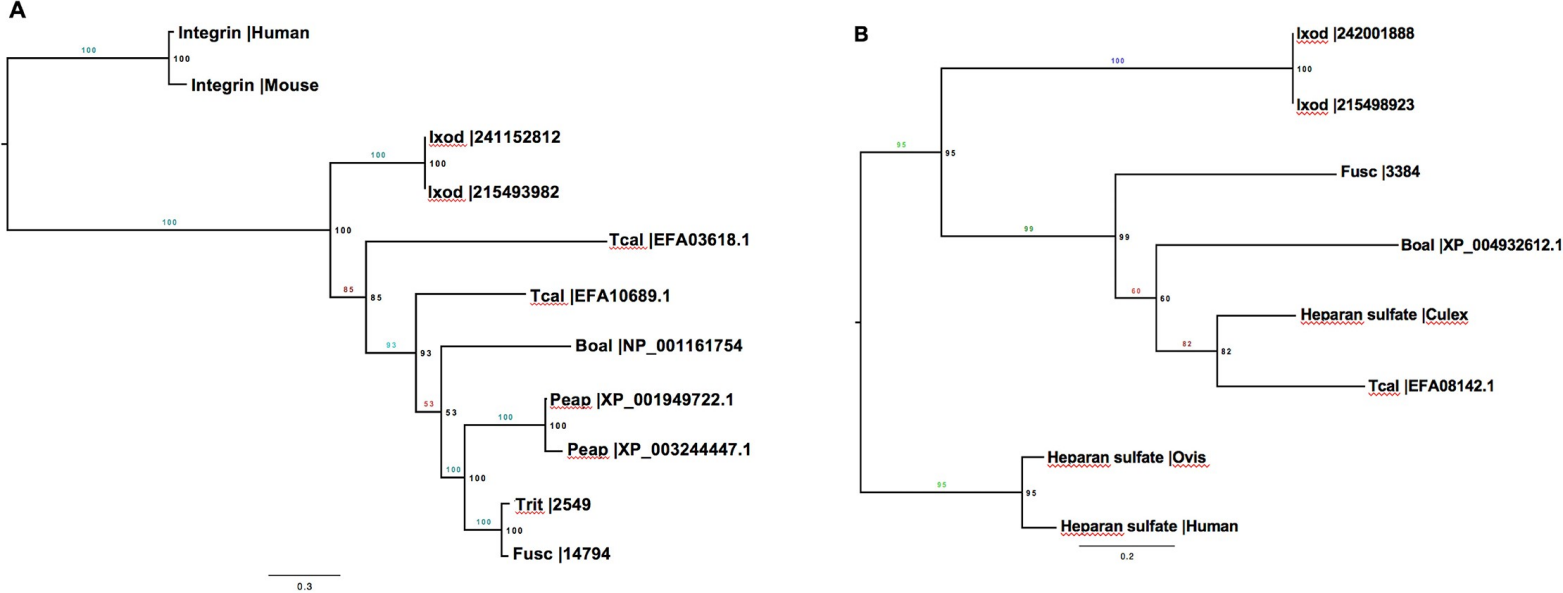
Phylogenetic analysis of integrin and heparan sulfate sequences. Protein sequences of integrin (A) and heparan sulfate (B) from *Homo sapiens* (Human), *Mus musculus* (Mouse), *Tribolium castaneum (Tcal)*, *Acyrthosiphon pisum* (Peap), *Ixodes scapularis* (Ixod), *Bombyx mori* (Boal), *Aedes albopictus* (Aedes), *Culex quinquefasciatus* (Culex), *Frankliniella fusca* (Fusc), and *Frankliniella tritici* (Trit) were used to construct phylogenetic trees. Phylogenetic trees were constructed using Randomized Axelerated Maximum Likelihood (RAxML) program in CIPRES software.

*Frankliniella fusca* and *Frankliniella tritici* transcriptomes were examined for Known receptors of animal-infecting bunyavirales’ members. Using OrthoMCL and phylogenetic analysis, homologs of integrin were identified in *F*. *fusca* and *F*. *tritici* “Fig 4A”. However, the homologs of heparan sulfate were only identified in *F*. *fusca* “Fig 4B”. Homologs of other bunyavirales receptors such as DC-SIGN and nucleolin were not present in either thrips species in this study.

### Viral infection related contigs in *F*. *fusca* and *F*. *tritici*

Blast2go annotated several contigs under the GO term “viral process” in *F*. *fusca* and *F*. *tritici*. The GO term viral process consisted of contigs associated with various processes of virus infection such as viral attachment, viral replication, and virus assembly. In *F*. *fusca*, 41 contigs were assigned to viral process “Table 2A”, while 31 contigs were assigned to viral process in *F*. *tritici* “Table 2B”. Some of the homologs of proteins present in both thrips species included ankyrin repeat domain-containing protein 17, host cell factors, heat shock protein 70, transcription elongation factor, and serine arginine-rich protein specific kinase 1b. The contigs of common homologs between two species were 88.1± 0.62% (Mean ± SE) similar. There were substantial number of polymorphisms that exist between the two species, it is not clear what these polymorphisms represent at this juncture. Further, homologs of several proteins specific to each thrips species also were identified. Out of 41 viral process contigs, 19 contigs including creb-binding protein, transcription initiation factor, transcriptional activator, and mitogen-activated protein kinase were only present in *F*. *fusca*. Eight homologs of proteins including nucleoporin seh1, radical s-adenosyl methionine domain containing protein, Ras-specific GTPase-activating protein, and Ras-related c3 botulinum toxin substrate were specific to *F*. *tritici*.

**Table 2:** Contigs associated with the Gene ontology term “Viral process”.

A. *Frankliniella fusca* contig ID	Annotation of contigs*
comp10366_c0_seq1	**26s proteasome non-ATPase regulatory subunit 12**
comp265632_c0_seq1	**60s ribosomal protein l31**
comp151775_c0_seq1	**60s ribosomal protein l31**
comp147082_c0_seq1	**60s ribosomal protein l5-like**
comp420931_c0_seq1	**A chain structural basis of the 70-kilodalton**
comp49158_c0_seq1	Ankyrin repeat domain-containing protein 17
comp10412_c0_seq1	Ap-2 complex subunit sigma
comp2417_c0_seq1	**ATP-dependent RNA helicase wm6-like**
comp28687_c0_seq1	**Creb-binding protein**
comp136931_c0_seq1	**Creb-binding protein**
comp55082_c0_seq1	**Creb-binding protein**
comp14610_c0_seq1	Cullin-1
comp4409_c0_seq3	Cullin-5
comp21018_c0_seq1	Cyclin t
comp19926_c0_seq1	DNA damage-binding protein 1
comp19735_c0_seq1	DNA-directed rna polymerase ii subunit rpb7
comp265420_c0_seq1	**DNA-directed RNA polymerases and iii subunit rpabc1**
comp11300_c0_seq1	**DNA-directed RNA polymerases and iii subunit rpabc4-l**
comp11034_c0_seq1	DNA-directed RNA polymerases and iii subunit rpabc5
comp13833_c0_seq1	**Double-stranded RNA-specific editase Adar**
comp3895_c0_seq1	HCFC 1 protein
comp190249_c0_seq1	Heat shock protein 70
comp38249_c0_seq1	**Mitogen-activated protein kinase 1**
comp4615_c3_seq3	Mothers against decapentaplegic homolog 3
comp20985_c0_seq1	Messenger RNA-capping enzyme
comp29498_c0_seq1	Nuclear cap-binding protein subunit 1
comp9368_c0_seq1	Proteasome subunit alpha type-4
comp11059_c0_seq1	Proteasome subunit beta type-3
comp3934_c0_seq1	Protein malvolio-like
comp74248_c0_seq1	**Ribosomal protein s10**
comp4966_c0_seq1	Ribosomal protein s13
comp4875_c0_seq1	Ribosomal protein ubq l40e
comp72127_c0_seq1	Serine arginine-rich protein specific kinase 1b
comp209984_c0_seq1	**Serine threonine-protein kinase**
comp729421_c0_seq1	**Structural protein vp1**
comp11384_c0_seq1	Transcription elongation factor b polypeptide 1
comp46336_c0_seq1	**Transcription initiation factor TFIID subunit 1**
comp250194_c0_seq1	**Transcriptional activator**
comp3297_c1_seq1	Transportin-1 isoform 1
comp2444_c0_seq1	Ubiquitin c
comp8398_c0_seq1	**zgc:172187 protein**
**B. *Frankliniella tritici* contig ID**	Annotation of contigs*
comp3007_c0_seq1	**40s ribosomal protein s27-like**
comp19953_c0_seq1	Ankyrin repeat domain-containing protein 17
comp7836_c0_seq1	Ap-2 complex subunit sigma
comp16066_c0_seq1	Cullin-1
comp42729_c0_seq1	Cullin-5
comp24747_c0_seq1	Cyclin t
comp2410_c1_seq1	DNA damage-binding protein 1
comp21095_c0_seq1	DNA-directed RNA polymerase ii subunit rpb7
comp13095_c0_seq1	DNA-directed RNA polymerases and iii subunit rpabc5
comp33688_c0_seq1	**E1a binding protein p300**
comp2994_c0_seq1	Heat shock protein 70
comp42177_c0_seq1	Host cell factor protein
comp63976_c0_seq1	Host cell factor protein
comp31317_c0_seq1	Mothers against decapentaplegic homolog 3
comp35513_c0_seq1	Messenger RNA-capping enzyme
comp18837_c0_seq1	Nuclear cap-binding protein subunit 1-like
comp27957_c0_seq1	**Nucleoporin seh1-a-like**
comp2418_c0_seq2	**Peptidyl-prolyl cis-trans isomerase e**
comp6226_c0_seq1	Proteasome subunit alpha type-4
comp6446_c0_seq1	Proteasome subunit beta type-3
comp18931_c0_seq1	Protein malvolio
comp1976_c0_seq1	**Radical S-adenosylmethionine**
comp13621_c0_seq1	**Ran-specific gtpase-activating protein**
comp1642_c0_seq1	**Ras-related c3 botulinum toxin substrate 1**
comp3081_c0_seq1	Ribosomal protein s13
comp1450_c0_seq1	Ribosomal protein ubq l40e
comp74095_c0_seq1	Serine arginine-rich protein specific kinase 1b
comp6684_c0_seq1	Transcription elongation factor b polypeptide 1
comp49373_c0_seq1	Transportin-1 isoform 1
comp2945_c0_seq1	Ubiquitin c
comp10867_c0_seq1	**UV excision repair protein rad23 homolog**

Contigs associated with the GO term “Viral process” in *Frankliniella fusca* (A) and *Frankliniella tritici* (B) are presented in the table.

*Contigs specific to each thrips species identified in this study are in bold.

### Immune genes in *F*. *fusca* and *F*. *tritici*

Using OrthoMCL, homologs of immune genes associated with pathogen pattern recognition, signal modulation, signaling pathways, and pathogen-suppressing molecules were identified in *F*. *fusca* and *F*. *tritici*. To confirm the presence of immune genes in *F*. *fusca* and *F*. *tritici*, homologs of immune genes from thrips species were aligned with best matched immune gene sequences from other arthropods and phylogenetic trees were constructed. Phylogenetic analysis confirmed the presence of 95 and 89 immune genes in *F*. *fusca* and *F*. *tritici*, respectively, and the data are presented in “Table 3”. Phylogenetic trees constructed on homologs of multigene families associated with immune pathways are presented as supplementary information (S1 File). Under pathogen pattern recognition, thirty-one homologs of immune genes associated with pathogen pattern recognition encoding peptidoglycan recognition protein (PGRP) “Figure A in S1 File”, scavenger receptor (SCR) “Figure B in S1 File”, and C-type lectin (CTL) “Figure C in S1 File” were identified in *F*. *fusca*, while 29 homologs of pattern recognizing proteins were identified in *F*. *tritici*. Immune genes such as clip domain serine proteases (CLIP) and serine protease inhibitors (Serpins) that are associated with signal modulation were also examined. The number of homologs of CLIP “Figure D in S1 File” was more in *F*. *fusca* (24) than in *F*. *tritici* (20). Four homologs of serpin “Figure E in S1 File” were identified in both *F*. *fusca* and *F*. *tritici*.

Most of the immune genes under the Toll pathway that were identified in *F*. *fusca* were also present in *F*. *tritici* “Figure F in S1 File”. However, homologs of Tollip were absent in *F*. *tritici*. Under IMD pathway, six and eight homologs of immune genes were identified in *F*. *fusca* and *F*. *tritici*, respectively. Several IMD pathway related immune genes such as Dredd, transforming growth factor b activated kinase (TAK), and Tak1-binding protein 2 (Tab2) were present in *F*. *tritici* but absent in *F*. *fusca*. In *F*. *tritici*, homologs of UbC13 were not present. Three homologs of JNK pathways related immune genes were identified in *F*. *fusca*, while in *F*. *tritici* two homologs were present. All the immune genes under JAK/STAT pathway: Domeless, STAT, and SOCs were present in both thrips species. Also, homologs of RNA interference (RNAi) associated immune genes including dicer and argonaute were present in *F*. *fusca* and *F*. *tritici*.

The presence of pathogen suppressing molecules that are activated by signaling pathways such as Toll, IMD, and JNK was examined. Pathogen suppressing molecules (antimicrobial peptides and enzymes) including prophenoloxidase “Figure G in S1 File”, Nitric oxide synthase (NOS), lysozyme, and caspase were identified in both thrips species. In *F*. *fusca*, nine homologs of pathogen suppressing molecules were identified, while in *F*. *tritici*, 10 homologs of pathogen suppressing molecules were identified.

**Table 3:** Immune gene homologs in *Frankliniella fusca* and *Frankliniella tritici*.

Immune gene families	*F*. *fusca*	*F*. *tritici*
**Pathogen pattern recognition**		
Peptidoglycan recognition proteins (PGRP)	4	3
beta-glucan recognition protein (beta-GRP)	2	2
Fibrinogen-related proteins (FRP)	1	1
Scavenger receptors (SCR)	11	11
C-type lectin (CTL)	6	5
Galectin	2	2
Thioester-containing protein (TEP)	1	2
Nimrod	1	1
Down syndrome cell adhesion molecule (Dscam)	3	2
**Signal modulation**		
Clip domain serine proteases (CLIP)	26	22
Serine protease inhibitors (Serpin)	4	4
**Signaling pathways**		
**A) Toll pathway**		
Toll	2	2
Spatzel	2	2
Tollip	2	0
Pellino	1	1
Pelle	1	1
TNF receptor-associated factor (TRAF)	1	1
**B) IMD pathway**		
Immune deficiency (IMD)	1	1
Dredd	0	1
Transforming growth factor b activated kinase (TAK)	0	1
Tak1-binding protein 2 (Tab2)	0	1
Inhibitor of apoptosis proteins (IAP2)	1	1
IKKB	2	2
UbC13	1	0
Relish	1	1
**C) JNK pathway**		
JNK	1	1
Fos	1	0
Jun	1	1
**D) JAK/STAT pathway**		
Domeless	1	1
Suppressor of cytokine signaling (SOCs)	1	1
STAT	1	1
**E) RNAi pathway**		
Dicer	2	2
Argonaute	2	2
**Pathogen suppressing molecules**		
Prophenoloxidase	4	4
Lysozyme-like protein	2	3
Nitric oxide synthase (NOS)	1	1
Defensin	1	0
Catalase	1	1
Caspase	0	1

Number of immune genes identified in *Frankliniella fusca* and *Frankliniella tritici* under three immune gene groups: pathogen recognition, signal modulation (including signaling pathways), and pathogen suppressing molecules. A database of well-annotated immune genes from arthropods including *Tribolium castaneum*, *Acyrthosiphon pisum*, *Drosophila melanogaster*, and *Bombyx mori* was created, and sequence match of these immune genes were identified in *F*. *fusca* and *F*. *tritici* using OrthoMCL. To confirm immune genes in *F*. *fusca* and *F*. *tritici*, phylogenetic trees were constructed with Randomized Axelerated Maximum Likelihood (RAxML) program using CIPRES software.

## Discussion

In this study, we performed qualitative comparisons of *F*. *fusca* and *F*. *tritici* transcriptomes. The functional annotations for the contigs in both transcriptomes were assigned into three categories. In the cellular component category, analysis of functional annotations revealed that they were mostly similar except for the GO terms cell envelope and external encapsulating structure, which were unique to *F*. *fusca*. Cell envelope and the external encapsulating structure could constitute the membrane complex in thrips cells. Cell membrane structures are particularly useful for plant-infecting enveloped viruses such as tospoviruses and rhabdoviruses to acquire their own envelopes and enter the host cells through fusion [36, 37]. It is interesting to find that these host factors are abundant in a vector as opposed to a non-vector. The role of these structures in virus transmission by thrips deserves further scrutiny. Both thrips species had similar functional groups across the biological process and molecular function categories. Analysis of biochemical pathways revealed that most of the pathways were associated with metabolism of lipids, carbohydrates, and amino acids. We further examined whether molecular factors associated with virus-vector interactions including virus reception, virus infection, and innate immunity varied between vector and non-vector thrips. *Frankliniella fusca* and *F*. *tritici* transcriptomes included homologs of the cell surface receptor integrin, while homologs of another receptor, heparan sulfate, were only present in *F*. *fusca*. Heparan sulfate is a heavily sulfated polysaccharide that is found pervasively on the cell surface and in the extracellular matrix of animal tissues [38]. The ubiquitous presence of heparan sulfate has allowed numerous viruses, bacteria, and other parasites to use heparan sulfate as a cell surface adhesion receptor to bind and gain entry in to host cells [39, 40]. Numerous mammalian DNA and RNA viruses (enveloped or non-enveloped) that do not require arthropod vectors such as members of herpesvirales, monengavirales, ortervirales, and papillomoviridae, and arthropod-borne viruses such as members of bunyavirales and flaviviridae also use heparan sulfate to enter host cells [41–50]. Studies have revealed that viruses have established close relationships with heparan sulfate based on its polysaccharide structure thereby allowing heparan sulfate to serve as a specific receptor [39]. It is interesting to find homologs of a robust and ubiquitous receptor such as heparan sulfate in vector thrips but not in non-vector thrips. Future studies should further examine in detail the role of heparan sulfate in the transmission of TSWV by thrips. Enzymatic removal of heparan sulfate has been specifically shown to reduce the binding of an enveloped RNA virus, rabies virus [50]. Such an approach or dsRNA mediated knockdown assay will help assess the importance of heparan sulfate as a cell surface adhesion receptor of TSWV in vector thrips.

In addition to virus receptors, we also identified several homologs of virus infection related proteins that were common and/or specific to *F*. *fusca* and *F*. *tritici*. In both thrips species, homologs of heat shock protein 70-kDa that is known to be involved in transcription and replication of influenza virus A [51, 52], and ankyrin repeat protein that is important for replication of myxoma virus [53] were identified. Homologs of mitogen activated protein kinase 1 that facilitates cellular entry of hepatitis C virus [54], and ribosomal protein s10 that interacts with human immunodeficiency virus [55], were present in *F*. *fusca* alone. In *F*. *tritici*, we identified homologs of GTPase- activating protein that facilitates replication of hepatitis C virus and sindbis virus [56, 57], serine arginine-rich protein specific kinase 1b that leads to phosphorylation of hepatitis B virus core protein [58], and nucleoporin protein that facilitates attachment of herpes simplex virus capsid protein to host nuclear complex [59]. Homologs of radical s-adenosylmethionine, an enzyme superfamily known to inhibit replication of several DNA and RNA viruses, were present only in *F*. *tritici*. The most well-studied example in this family is Viperin–*virus inhibitory protein*, *endoplasmic reticulum-associated*, *interferon inducible* [60]. Infection in humans and other animals by numerous viruses is characterized by upregulation of Viperin [61]. In influenza virus, radical s-adenosylmethionine domain containing Viperin is known to disrupt cholesterol rich lipid rafts that are used in virus budding off from the plasma membrane [62–64]. Viperin has also been known to suppress the multiplication of an enveloped rabies virus by targeting cholesterol and sphingomyelin production [65]. In addition to inhibiting virus budding, in the lentivirus, equine infectious anemia virus, Viperin is known to inhibit the release of viral group specific antigen (Gag) and envelop coding protein (Env), and disrupt virus receptors [66]. These studies suggest that virus suppression by radical s-adenosylmethionine domain containing Viperin could occur in multiple ways. The functional characterization of Viperin is accomplished either by correlating upregulation of Viperin with reduced virus multiplication or by blocking Viperin transcription and correlating it to enhanced virus multiplication [66]. The presence of an important virus multiplication inhibitor such as radical s-adenosylmethionine in the non-vector (*F*. *tritici*) and absence in the vector (*F*. *fusca*) offers substantive insights on virus-vector interactions. As mentioned earlier, TSWV is enveloped with glycolipids, and is similar to many animal infecting members of bunyavirales. Studies on the role of radical s-adenosylmethionine in TSWV transmission should be undertaken to assess if this enzyme affects thrips ability to function as vector of TSWV.

Homologs pertaining to several immune genes were found in *F*. *fusca* and *F*. *tritici* transcriptomes. Most of the immune genes associated with major antiviral pathways including RNAi, Toll, and JAK/STAT were present in both thrips species. The IMD pathway has been demonstrated to have antiviral activity in *D*. *melanogaster* [67, 68]. The loss of IMD pathway related immune genes in *D*. *melanogaster* cell lines increased cricket paralysis virus (CrPV) load and enhanced sensitivity to CrPV infection. Several IMD pathway related upstream immune genes including Dredd, TAK, Tab2 were present in *F*. *tritici* but absent in *F*. *fusca*. In *Drosophila* and other insects, Dredd is required to cleave Imd, leading to ubiquination by Iap2 and subsequent binding and activation of the Tab2/Tak1 complex, resulting in phosphorylation and activation of the IKK complex [69]. The absence of Dredd, TAK, and Tab2 in *F*. *fusca* suggests that the IMD pathway could be disabled in *F*. *fusca*. It is not clear whether the lack of IMD related immune genes is specific to *F*. *fusca* or in multiple vector thrips species. More studies need to be conducted to examine the importance of IMD pathway in virus suppression by vector and non-vector thrips.

Qualitative comparison between *F*. *fusca* and *F*. *tritici* transcriptomes revealed several important differences between vector and non-vector thrips species in terms of virus reception, virus infection, and innate immunity. A majority of contigs associated with virus-vector interaction was present in both thrips species. However, some contigs such as homologs of heparan sulfate associated with virus entry were only present in *F*. *fusca*, homologs of radical s-adenosylmethionine that inhibits virus replication were specific to *F*. *tritici*, and several IMD pathway related immune genes’ homologs were absent in *F*. *fusca*. The role of these genes in TSWV transmission needs further examination. Nevertheless, identification of these genes suggests that vector and non-vector thrips species could differently interact with the virus. To our knowledge, this is the first study to compare transcriptomes of vector and non-vector thrips species. Transcriptomic data generated in this study were deposited into a public (National Center for Biotechnology Information—NCBI) database with Sequence Read Archive (SRA) accession numbers SRP023246 and SRP023248. *Frankliniella fusca* and *F*. *tritici* transcriptomes from this study could serve as important genomic resources for future studies on other vector and non-vector thrips species.

## Materials and methods

### Maintenance of thrips colony

A *F*. *fusca* colony was established in 2009 with thrips collected from peanut blooms at the Belflower Farm (Coastal Plain Experimental Station, Tifton, GA, USA) and identified using published morphological keys under a dissecting microscope (Leica MZ6) at 64 X magnification [70]. The identified adults were used for initiating the colony. Thrips were maintained in Munger cages (0.11 X 0.89 X 0.18 m) in a growth chamber (Thermo Scientific, Dubuque, IA, USA) at 25–30°C, 40–50% relative humidity, and L14:D10 photoperiod [71]. Thrips were reared on non-infected leaflets of the peanut cultivar, Georgia Green. The peanut plants were maintained in thrips-proof cages (Megaview Science, Taichung, Taiwan). *Frankliniella tritici* used for the study were collected from peanut fields at the Coastal Plain Experimental Station.

### Total RNA extraction, library preparation, and sequencing

Approximately, 35 non-virus exposed female *F*. *fusca* and field-collected *F*. *tritici* adult females were pooled separately for total RNA extraction. Total RNA was extracted using RNeasy mini kit using manufacturer’s protocol (Qiagen, Valencia, CA, USA). Subsequently, mRNAs (polyadenylated RNAs) were selected using oligo-dT and cDNA libraries were constructed at Georgia Genomic Facility of the University of Georgia. Prior to library construction, Agilent 2100 Bioanalyzer (Agilent Technologies, Santa Clara, CA, USA) was used to evaluate RNA quality and concentration of the samples. Illumina sequencing libraries were constructed using TruSeq RNA sample preparation kits using at least 1 μg of the total RNA. Messenger RNA was selected, fragmented, and then reverse transcribed into cDNA. Subsequently, second strand cDNA was prepared using Polymerase I and RNase H, and TruSeqLT adapters were ligated to the DNA fragments for PCR amplification. Finally, two libraries were sequenced on Illumina HiSeq 2000 platform using paired-end 100 cycle sequencing settings at the University of Texas Health Science Center, at San Antonio, Texas.

### Processing of the RNA-seq reads and transcriptome assembly

Raw RNA-seq reads were processed using bioinformatics software available at the Georgia Advanced Computing Resource Center, UGA (https://wiki.gacrc.uga.edu/wiki/Software). Adapter sequences were trimmed using the Trimmomatic software (Version 0.36) [72]. Trimmomatic was also used to produce quality reads by removing three bases at the beginning and end of each read, setting minimum read length threshold to 50 bases, and discarding reads if the average quality of four bases fell below 20. After cleaning reads, Trinity (Version 0.36) software was used to perform *de novo* assembly on *F*. *fusca* and *F*. *tritici* reads individually using the following parameters “–kmer 25 –minimum-contig-length 300 bp” [73]. The assembled contigs from each thrips species were subjected to CEGMA (Core Eukaryotic Genes Mapping Approach) program (Version 2.5) to assess the completeness of the assembly [74]. The completeness of the transcriptomes was also evaluated using another software (BUSCO, Version 3.0.2) [75].

### Functional annotations of *F*. *fusca* and *F*. *tritici* contigs

Assembled contigs from *F*. *fusca* and *F*. *tritici* were annotated using a java-based Blast2go software (Version 3.2) (https://www.blast2go.com) [76]. First, Blastx was used to search sequence similarity against the NCBI non-redundant protein database with E-value threshold of 10^−6^. Subsequently, Blast2go assigned Gene Ontology (GO) terms to each contig with an E-value of 10^−6^ and annotation cutoff of 55. GO terms were categorized under biological process, molecular process, and cellular component based on a node score of 5 and level 5. Biochemical pathways were assigned to *F*. *fusca* and *F*. *tritici* contigs using the KEGG database in Blast2go.

### Virus-vector interaction associated molecular factors

Following functional annotations of *F*. *fusca* and *F*. *tritici* contigs, we focused on molecular factors that could influence thrips ability to transmit TSWV including 1) virus receptors, 2) virus infection related proteins, and 3) immune genes in *F*. *fusca* and *F*. *tritici*.

### Virus receptors in *F*. *fusca* and *F*. *tritici*

We investigated known receptors of animal-infecting members of bunyavirales including integrin, heparan sulfate, nucleolin, and DC-SIGN in *F*. *fusca* and *F*. *tritici* using OrthoMCL software (Version 2.0.9) [77]. First, well annotated receptor sequences from humans, *Homo sapiens* [L.], mouse, *Mus musculus* [L.], sheep, *Ovis aries* [L.], southern house mosquito, *Culex quinquefasciatus* [Say], and Asian tiger mosquito, *Aedes albopictus* [Skuse] were gathered and a database was created [32, 33, 78]. Sequences from other arthropods including red flour beetle, *Tribolium castaneum* [Herbs], pea aphids, *Acyrthosiphon pisum* [Harris], deer tick, *Ixodes scapularis* [say], and silkworm, *Bombyx mori* [L.] were also downloaded from the National center for biotechnology information and added to the database. The receptor sequences were used as query sequences and their homologs were identified in *F*. *fusca* and *F*. *tritici* contigs generated in this study. First, using Blastp, sequence match of all the proteins were identified with an E-value of 10^−6^. Blast result was then filtered by setting threshold of percent match for each pair of sequences to 50%. The homologous sequences were grouped using MCL software, and phylogenetic trees were constructed to validate grouping of the sequences. Protein sequences from each group were aligned using a Multiple alignment using Fast Fourier Transform software (http://mafft.cbrc.jp/alignment/software/), and the alignment was manually cured in Mesquite (http://mesquiteproject.wikispaces.com/installation) [79]. Subsequently, phylogenetic analyses were performed using Randomized Axelerated Maximum Likelihood (RAxML) program with CIPRES (Version 8) [80], and trees were constructed using Fig Tree software (Version 1.4.3) [81] to confirm the presence of receptors in *F*. *fusca* and *F*. *tritici*. For this analysis, protein/AA data type was chosen, and a JTT protein substitution matrix for analysis was selected. Boot strapping analysis was conducted to search for the best-scoring maximum likelihood tree. Based on software recommendations, bootstrapping was halted automatically. The RaxML generated tree was subsequently opened in FigTree software, and node and tip labels were added. Only sequences in the trees with the node score of more than 60 were considered for the study.

### Immune genes in *F*. *fusca* and *F*. *tritici*

Homologs of immune genes were also identified in *F*. *fusca* and *F*. *tritici* using the OrthMCL software. A database of well-annotated immune genes from arthropods including *T*. *castaneum* [82], *A*. *pisum* [83], fruit fly *Drosophila melanogaster* [Fallen] [21], and *B*. *mori* [84] was created, and sequence match of the immune genes was identified in *F*. *fusca* and *F*. *tritici* as described previously using OrthoMCL. To validate the clustering of immune genes, homologs of immune genes from the thrips species along with their best matched immune gene sequences from the arthropod species identified in OrthoMCL were aligned, and phylogenetic trees were constructed with the RAxML program using CIPRES software as described previously. Phylogenetic trees of multigene immune families are provided as supplementary materials. Identified immune genes were categorized into three gene groups namely pathogen recognition, signal modulation (including signaling pathways), and pathogen suppressing molecules.

## Supporting information

S1 File Phylogenetic analysis of immune genes in *Frankliniella fusca* and *Frankliniella tritici*. Protein sequences of immune genes from *Tribolium castaneum* (TC), *Drosophila melanogaster* (DM), *Bombyx mori* (BM), and *Acyrthosiphon pisum* (AP) along with their homologs identified in *Frankliniella fusca* (FF) and *Frankliniella tritici* (FT) through OrthoMCL were aligned and phylogeneic trees were constructed. Phylogenetic trees for peptidoglycan recognition protein “Figure A in S1 File”, scavenger receptor (SCR) “Figure B in S1 File”, C-type lectin (CTL) “Figure C in S1 File”, Clip domain serine proteases (CLIP) “Figure D in S1 File”, serpin “Figure E in S1 File”, Toll pathway “Figure F in S1 File”, and Prophenoloxidase “Figure G in S1 File” were constructed using Randomized Axelerated Maximum Likelihood (RAxML) program using CIPRES software.
(PPTX)

S1 Table KEGG pathway analysis in *Frankliniella fusca* and *Frankliniella tritici*. KEGG pathways assigned to *Frankliniella fusca* and *Frankliniella tritici* contigs using KEGG database in Blast2go.
(XLSX)
