# Supplementary figures and images for: Comparison of transcriptomes of an orthotospovirus vector and non-vector thrips species
Source: PLoS One. 2019 Oct 10;14(10):e0223438. doi: 10.1371/journal.pone.0223438 (PMC6786753; doi:10.1371/journal.pone.0223438)

## Slide 1
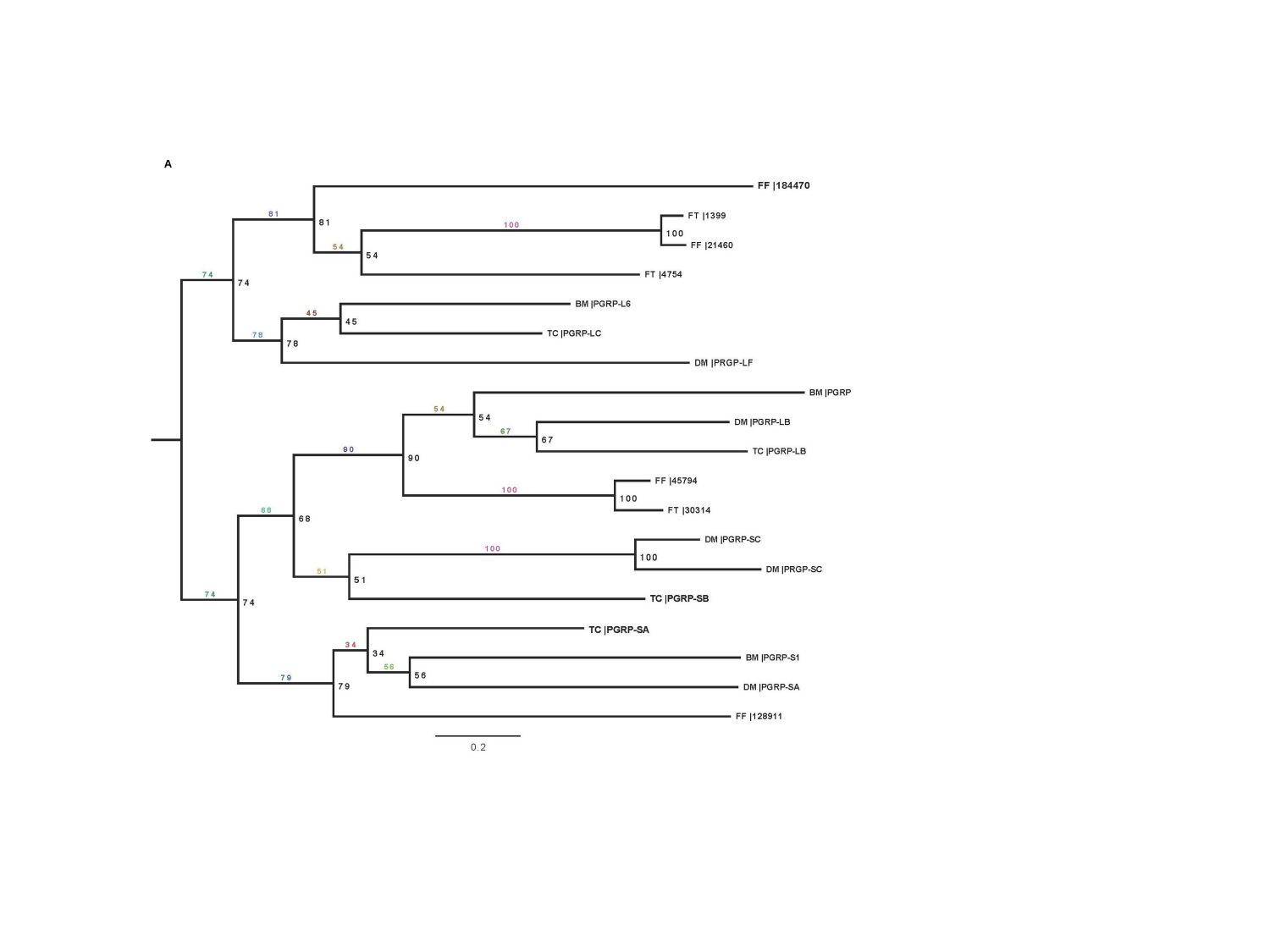

# A

## Slide 2
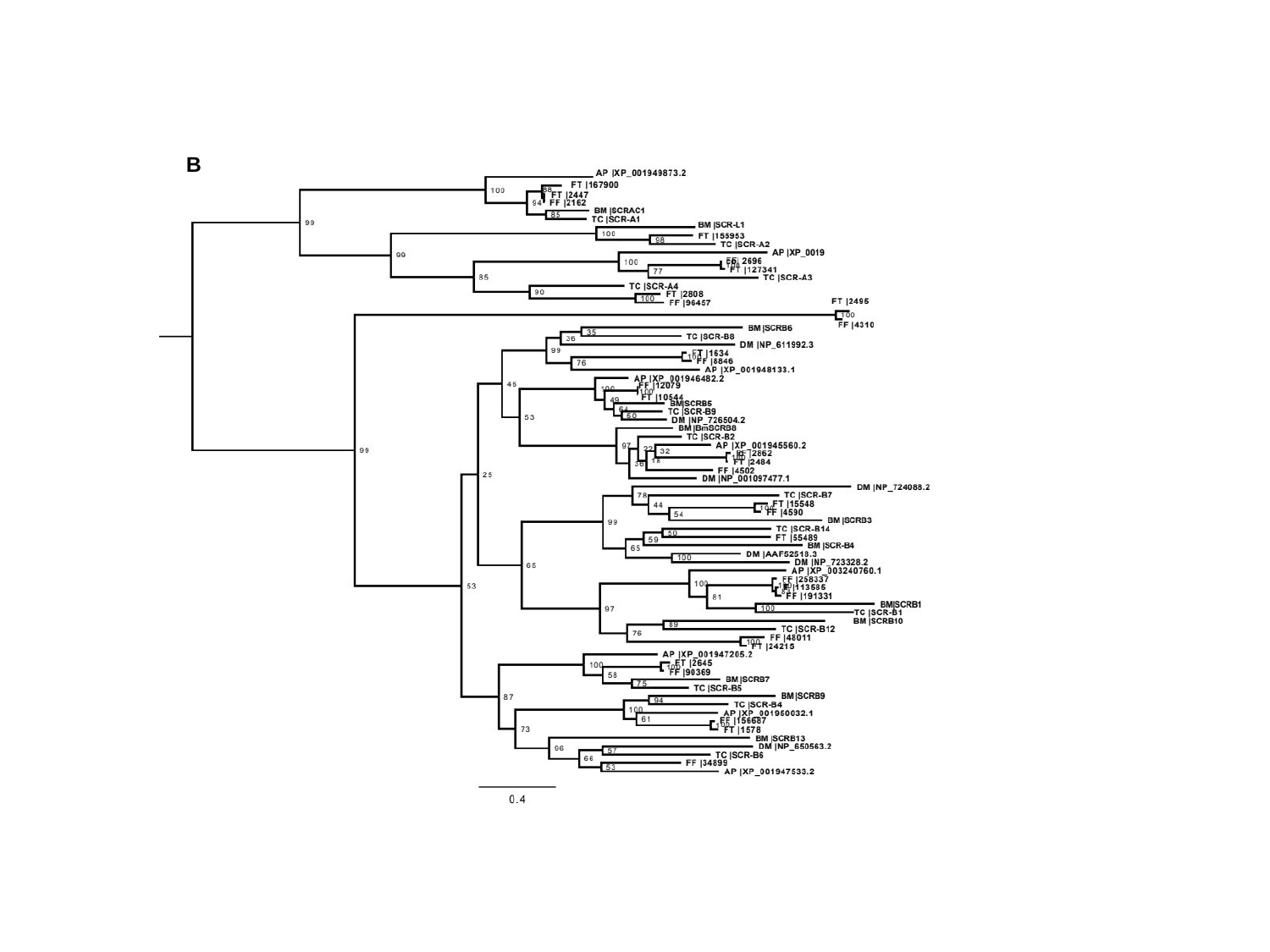

B

## Slide 3
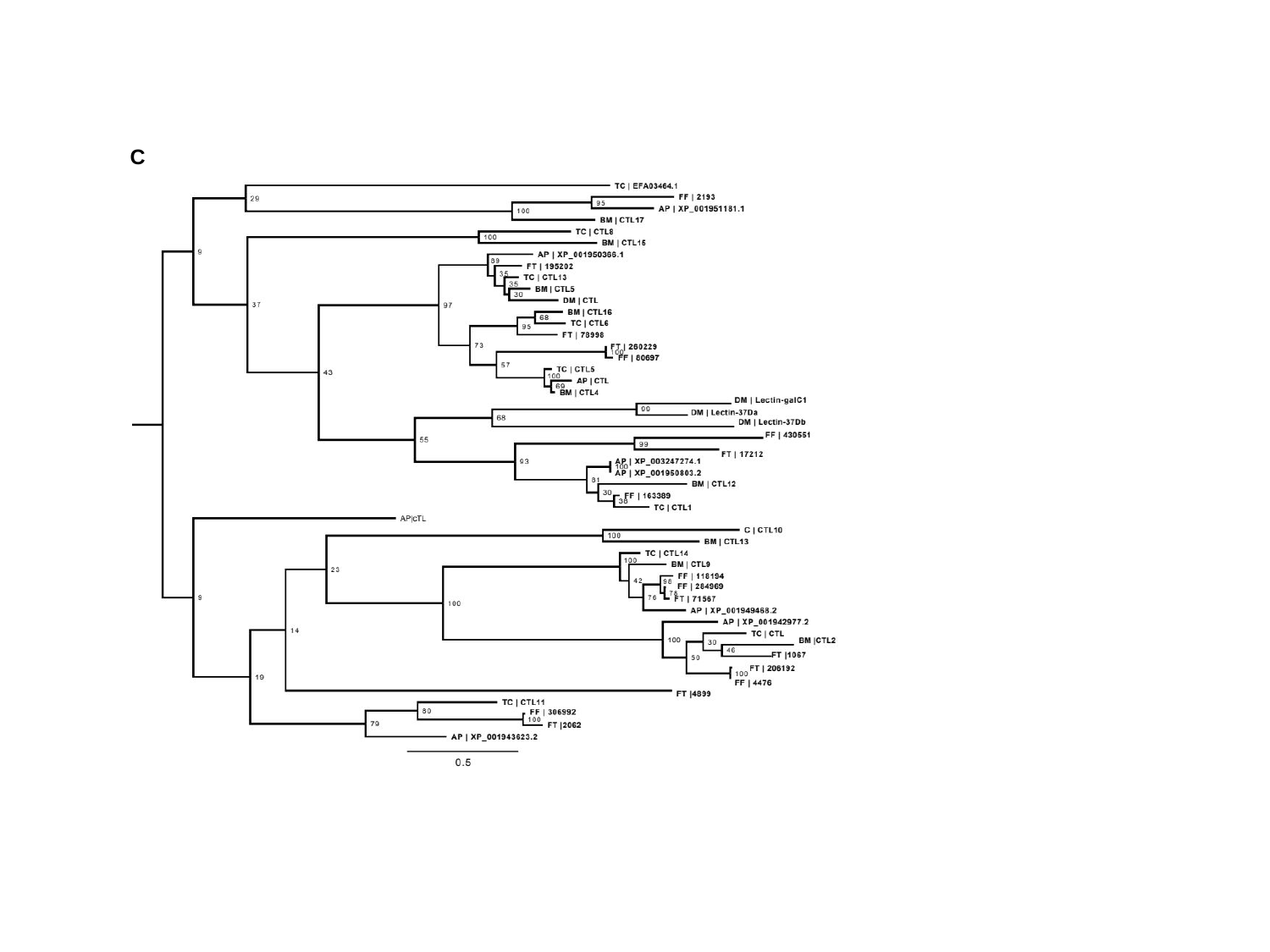

C

## Slide 4
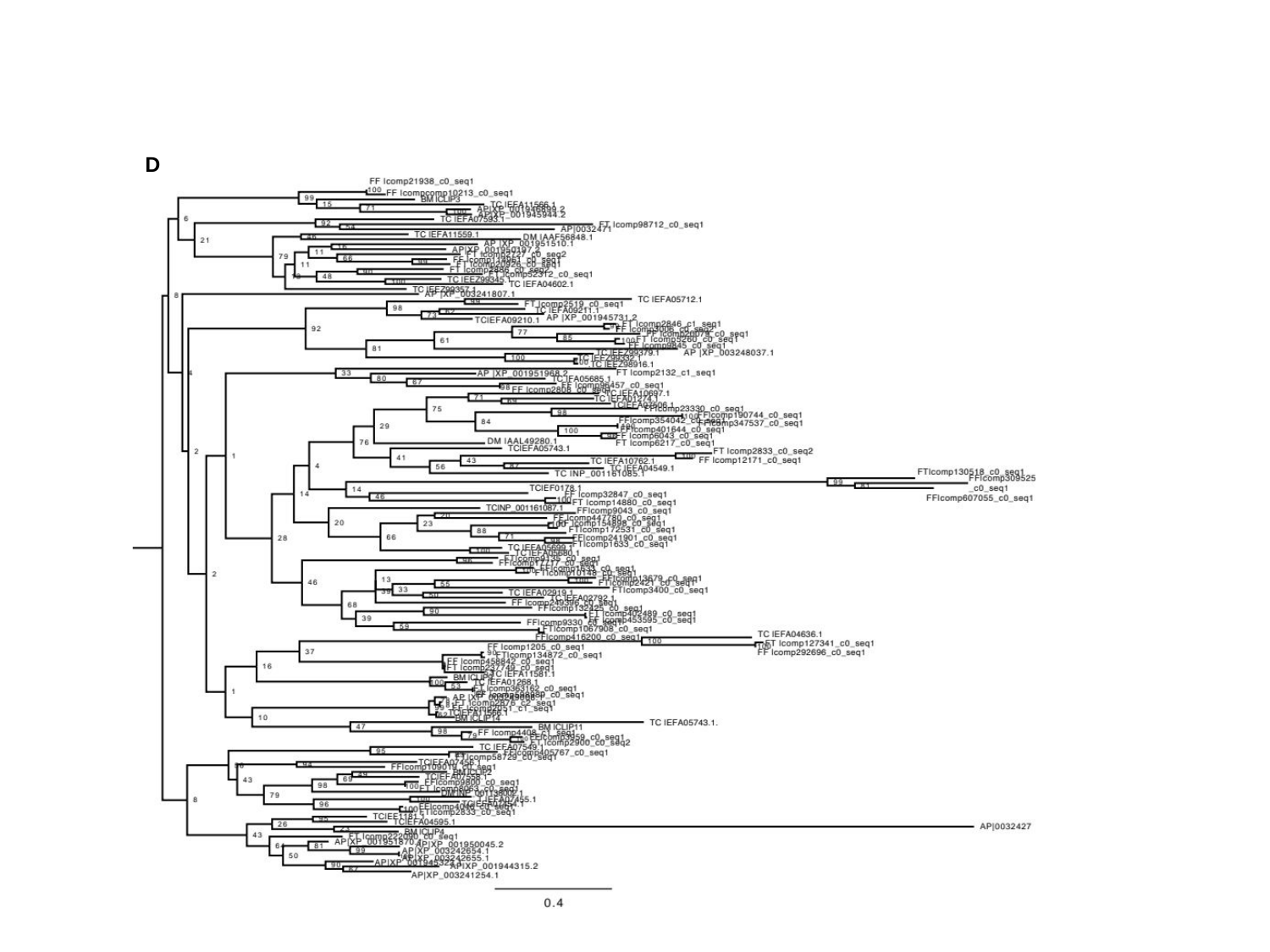

D

## Slide 5
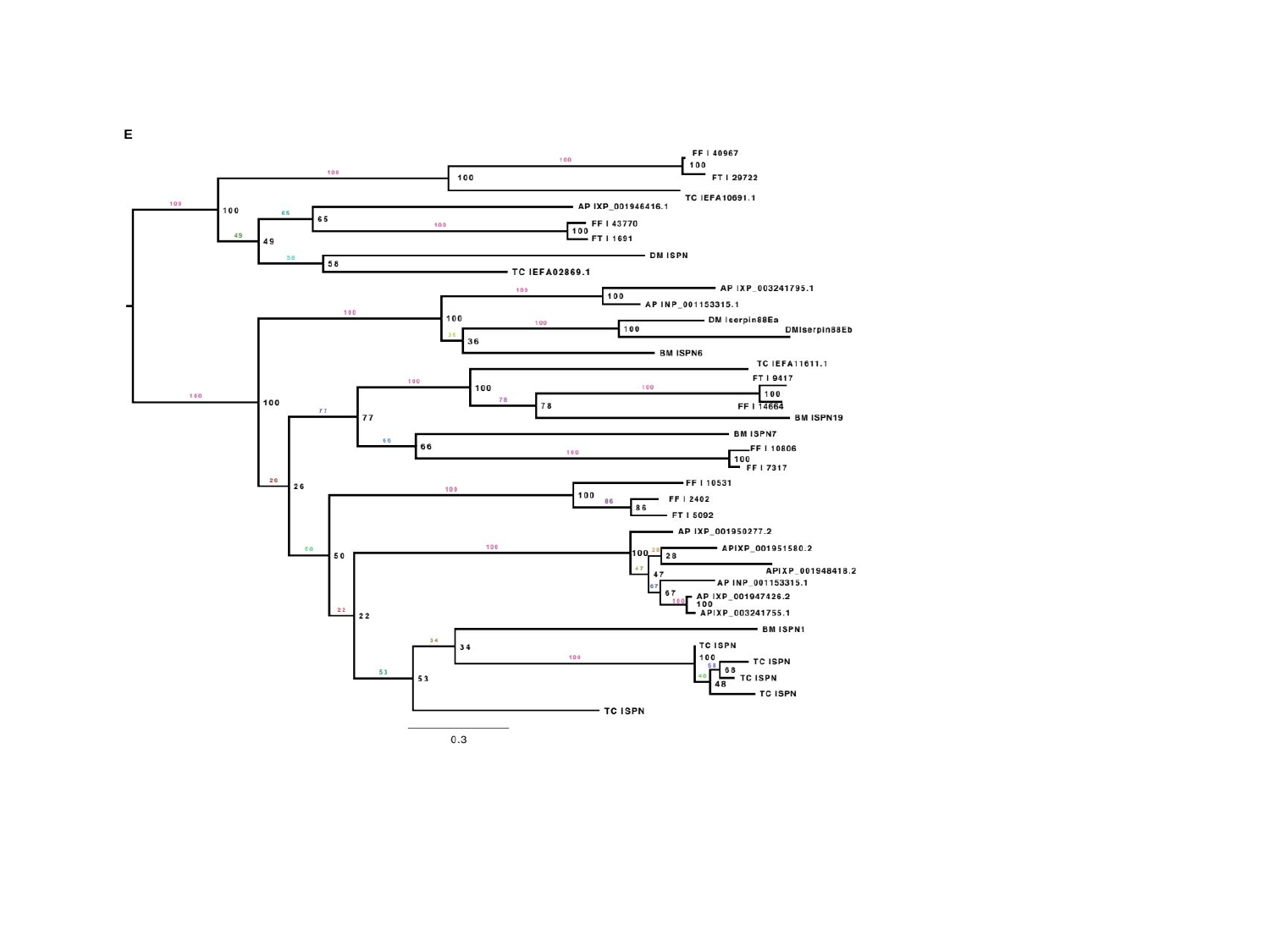

# E

## Slide 6
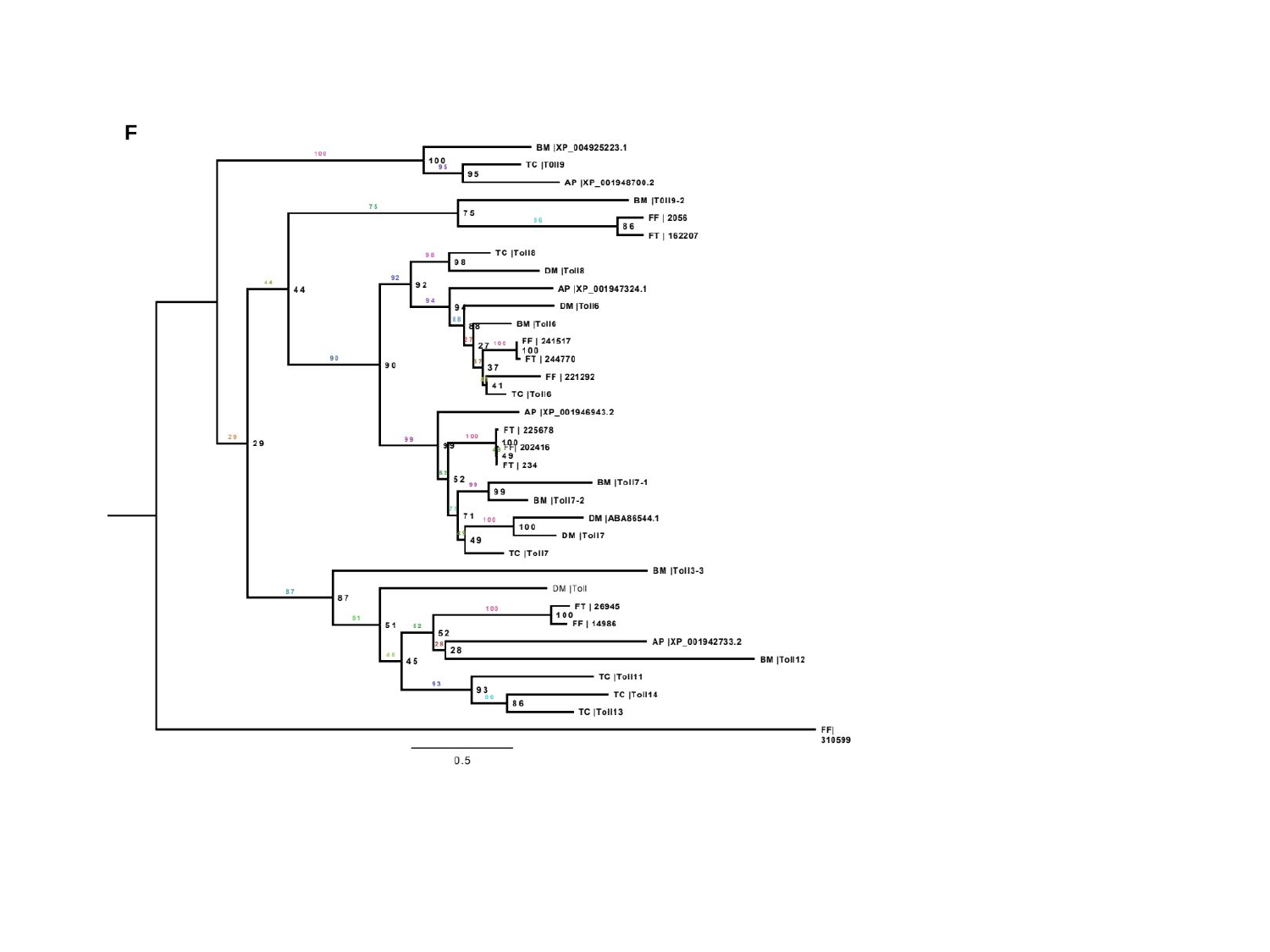

F

## Slide 7
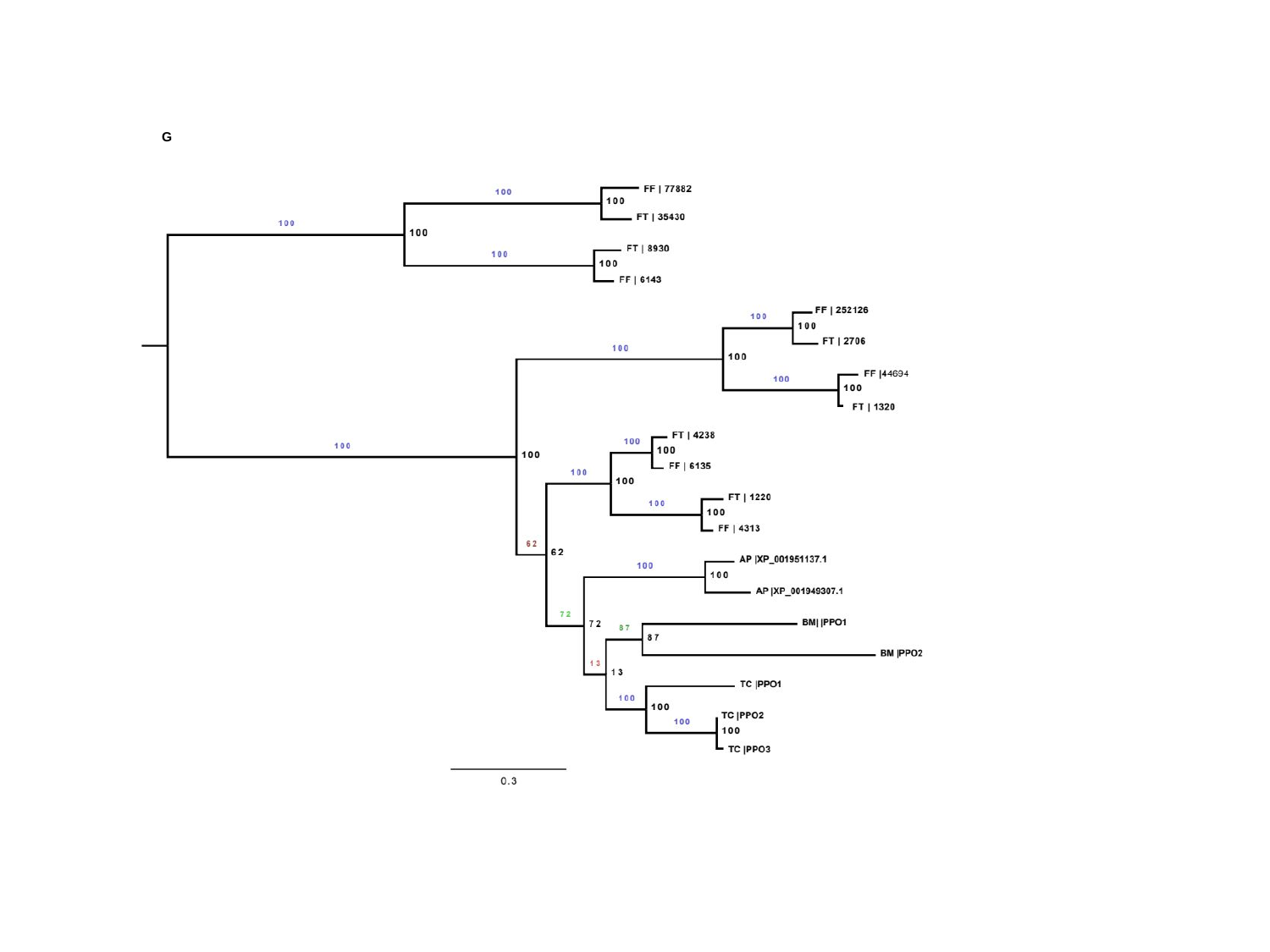

# G

Supplement: S1 File — Protein sequences of immune genes from Tribolium castaneum (TC), Drosophila melanogaster (DM), Bombyx mori (BM), and Acyrthosiphon pisum (AP) along with their homologs identified in Frankliniella fusca (FF) and Frankliniella tritici (FT) through OrthoMCL were aligned and phylogeneic trees were constructed. Phylogenetic trees for peptidoglycan recognition protein “Figure A in S1 File”, scavenger receptor (SCR) “Figure B in S1 File”, C-type lectin (CTL) “Figure C in S1 File”, Clip domain serine proteases (CLIP) “Figure D in S1 File”, serpin “Figure E in S1 File”, Toll pathway “Figure F in S1 File”, and Prophenoloxidase “Figure G in S1 File” were constructed using Randomized Axelerated Maximum Likelihood (RAxML) program using CIPRES software. (PPTX) [file pone.0223438.s001.pptx]
